# Supplementary material for: Seminoma and Embryonal Carcinoma Footprints Identified by Analysis of Integrated Genome-Wide Epigenetic and Expression Profiles of Germ Cell Cancer Cell Lines
Source: PLoS One. 2014 Jun 2;9(6):e98330. doi: 10.1371/journal.pone.0098330 (PMC4041891; doi:10.1371/journal.pone.0098330)
Supplement: File S1 — ZIP file containing DMRforPairs output for significant regions. Please start from the html files. (ZIP) [file pone.0098330.s008.zip › figures/35532.pdf]

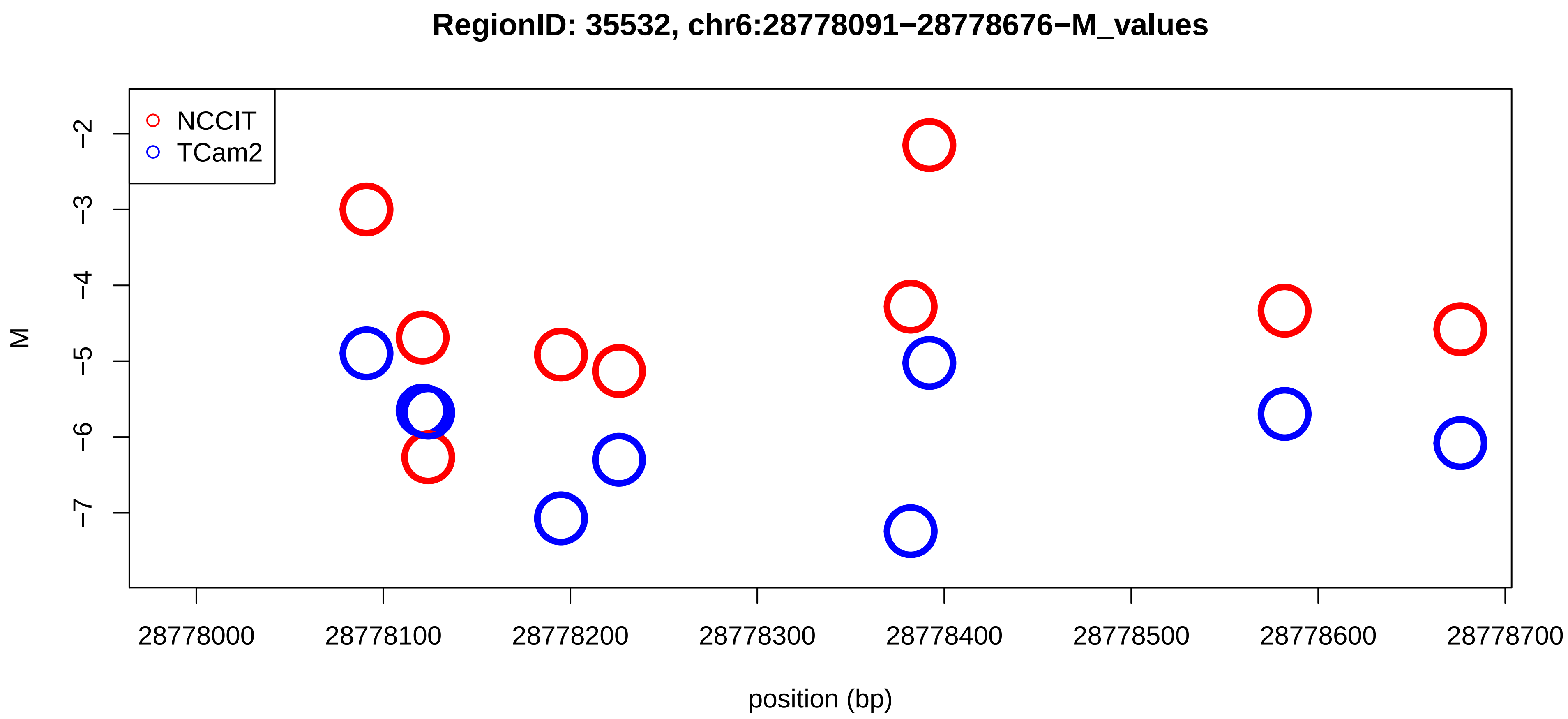

RegionID: 35532, chr6:28778091−28778676−M_values
NCCIT
TCam2
M
−2
−3
−4
−5
−6
−7
28778000
28778100
28778200
28778300
28778400
28778500
28778600
28778700
position (bp)

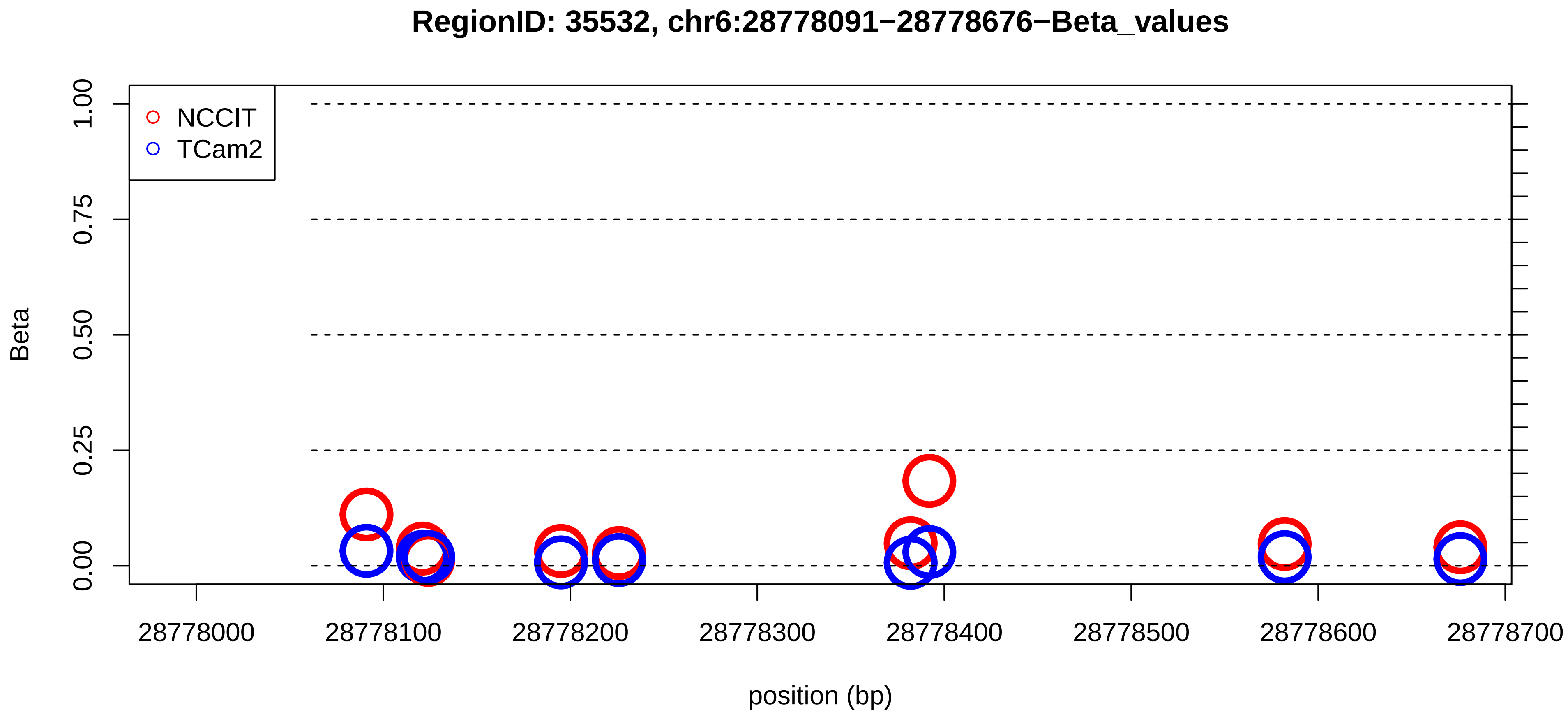

RegionID: 35532, chr6:28778091−28778676−Beta_values
NCCIT
TCam2
Beta
0.00
0.25
0.50
0.75
1.00
28778000
28778100
28778200
28778300
28778400
28778500
28778600
28778700
position (bp)
